# Energy-efficient biomass processing with pulsed electric fields for bioeconomy and sustainable development

**DOI:** 10.1186/s13068-016-0508-z

**Published:** 2016-04-27

**Authors:** Alexander Golberg, Martin Sack, Justin Teissie, Gianpiero Pataro, Uwe Pliquett, Gintautas Saulis, Töpfl Stefan, Damijan Miklavcic, Eugene Vorobiev, Wolfgang Frey

**Affiliations:** Porter School of Environmental Studies, Tel Aviv University, Tel Aviv, Israel; Institute for Pulsed Power and Microwave Technology, Karlsruhe Institute of Technology, Karlsruhe, Germany; CNRS, Institut de Pharmacologie et de Biologie Structurale Université de Toulouse, Toulouse, France; Department of Industrial Engineering, University of Salerno, via Giovanni Paolo II 132, 84084 Fisciano, SA Italy; Institut für Bioprozeβ- und Analysenmeβtechnik e.V., Heilbad Heiligenstadt, Germany; Department of Biology, Faculty of Natural Sciences, Vytautas Magnus University, Kaunas, Lithuania; German Institute of Food Technologies, Quakenbrück, Germany; Faculty of Electrical Engineering, University of Ljubljana, Ljubljana, Slovenia; Departement de Genie Chimique, Centre de Recherche de Royallieu, Universite de Technologie de Compiegne, Compiegne, France

**Keywords:** Biorefinery, Bioeconomy, Sustainable development, Pulsed electric fields, Electroporation, Electrobiorefinery

## Abstract

Fossil resources-free sustainable development can be achieved through a transition to bioeconomy, an economy based on sustainable biomass-derived food, feed, chemicals, materials, and fuels. However, the transition to bioeconomy requires development of new energy-efficient technologies and processes to manipulate biomass feed stocks and their conversion into useful products, a collective term for which is biorefinery. One of the technological platforms that will enable various pathways of biomass conversion is based on pulsed electric fields applications (PEF). Energy efficiency of PEF treatment is achieved by specific increase of cell membrane permeability, a phenomenon known as membrane electroporation. Here, we review the opportunities that PEF and electroporation provide for the development of sustainable biorefineries. We describe the use of PEF treatment in biomass engineering, drying, deconstruction, extraction of phytochemicals, improvement of fermentations, and biogas production. These applications show the potential of PEF and consequent membrane electroporation to enable the bioeconomy and sustainable development.

## Introduction to biorefineries for sustainable development and the need for new technologies

Food, chemicals, and industrial sectors are challenged with the growing population, increasing longevity and quality of life. The increasing demand in these major sectors of economy will increase the consumption of fossils energy sources, agricultural land, and drinking water. This demand could lead to the irreversible changes in climate with unpredictable consequences. A possible direction to address this challenge sustainably is increasing the efficiency of currently used processes and displacement of fossil fuels energy sources by production of useful biomass [1, 2]. This substitution of the fossil resources-derived chemicals and fuels with biomass for the production of food, platform chemicals, and fuels is known as bioeconomy [3]. A basic productive unit in the bioeconomy is biorefinery. Bringing biorefineries to practice is expected to contribute to low-carbon economies, by production of chemicals, energy, and jobs without using fossil fuels [4]. The design and implementation of biorefinery depend on a large number of factors, including availability of feedstocks, advances in the biomass production and processing technologies, environmental impacts, and socio-economic conditions [2, 5].

Despite the long history of biomass use by humans, biomass processing and converting technologies are mostly traditional and not efficient in terms of outputs and energy consumption. Therefore, fossil sources are often preferred for synthetic chemicals production and energy generation. During the last centuries, fossil fuels-based processes achieved very high efficiency (for e.g., ~35 % efficiency of electricity generation in the oil and coal power station and ~60 % in the combined gas turbines) [6]. Today biomass-based processes are fundamentally less efficient than fossil fuel sources as the efficiency of the solar energy conversion to chemical energy by photosynthesis is 5 % at most [7]. However, given the advantages of biomass in terms of low-carbon footprint, and, in some cases, low-water footprint, versatility of products, and local and global availability, there is a strong motivation to develop new processes and technologies that will boost the energy efficiency of biorefineries [8]. One type of these new technologies is based on pulsed electric fields (PEF). First, this technology was developed in the USSR in 1940s and 1950s [9, 10] and then in Europe in 1960s [11] for juices and phytochemicals extraction, and microorganisms inactivation [12]. Recent tremendous developments in the fundamental understanding on PEF impact on cells, development of new processes and technologies, suggest that PEF could become an essential tool for energy-efficient biorefineries [13–15].

**Fig. 1:**
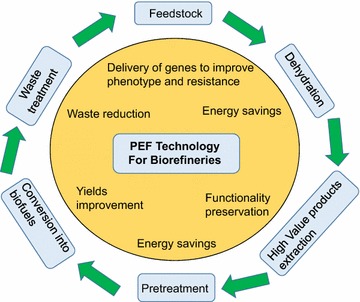
Applications of pulsed electric field (PEF) technologies for biorefineries. Pulsed electric field technology can find useful implementation in multiple processes in biorefinery. It can be used for gene transfection to improve feedstocks, save energy during drying and pretreatment, preserve functionality and specificity of the extracted high-value products, improve yields of the produced biofuels, and reduce wastes

The focus of this review is to present and critically discuss the use of PEF in biorefineries that enable bioeconomy (Fig. 1). First, it briefly describes the fundamentals of pulsed electric field-induced electroporation of biological cell membrane, as well as the technologies and devices at the pilot and industrial scale that are already used for biorefineries applications. Second, the review proceeds with the description of the several processes of the biorefinery that can be positively affected by the use of PEF technology. In particular, the use of PEF for (1) biorefineries feedstock development through gene electroporation, (2) biomass drying, (3) extraction of high added-value products from waste, lignocellulose biomass, and microalgae, (4) extraction of molecules from bacteria and yeast, and (5) biogas production, is discussed with more details. Finally, this paper also suggests next steps that should be followed to integrate PEF technology in the biorefinery networks (Fig. 1). This review is in part result of networking efforts within the COST TD1104 Action [15] and in particular of the workshop organized in Compiegne, France, Jan 27–28, 2015.

## Pulsed electric field technology fundamentals: the cell membrane electroporation phenomena

**Fig. 2:**
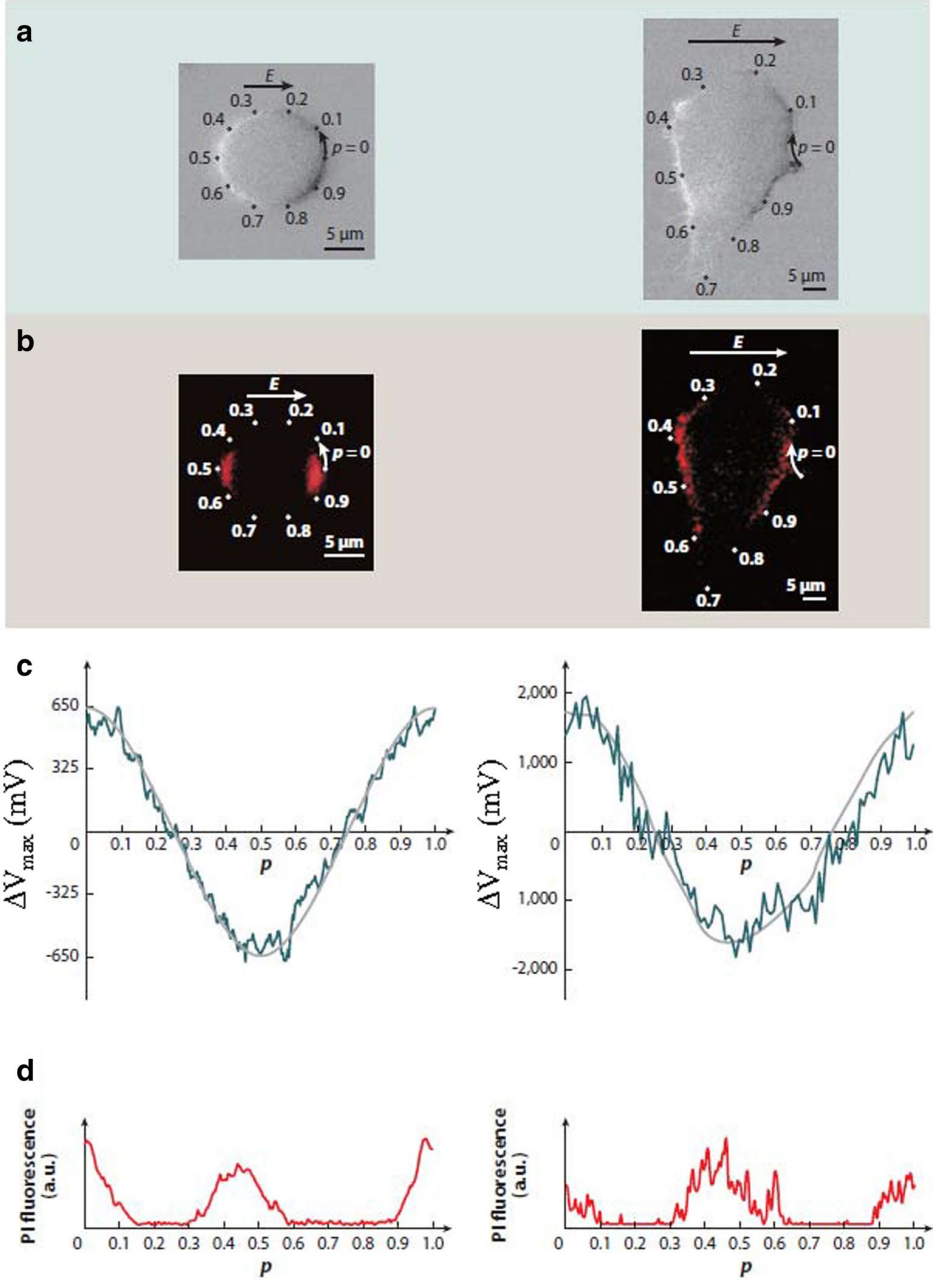
E.g., of the electric field-induced transmembrane voltage (TMV). Two Chinese hamster ovary (CHO) cells in a physiological medium were exposed to the electric fields. One cell has an almost spherical geometry (*left-hand sides of panels* **a**–**d**) and was suspended. This cell was exposed to non-porating single 50-ms, 100-V/cm pulse. The second cell is irregularly shaped and is attached (*right-hand sides of panels* **a**–**d**). This cell was electroporated by a single 200-μs, 1000-V/cm pulse. **a** Membrane depolarization and hyperpolarization as detected with changes in the fluorescence of di-8-ANEPPS, a potentiometric dye reflecting the TMV. *E* is the strength of the electric field, *p* is the axes of rotational symmetry of the cell. **b** PEF mediated influx into the cell as detected with fluorescent dye propidium iodide (PI) as imaged 200 ms after exposure. **c** Measured (*green*) and predicted by numerical computation (*gray*) TMV. **d** PI signal. P shows a normalized arc length along the membrane Figure adapted from [28], based on [158]

When a cell is exposed to PEF, additional transmembrane voltage (TMV, Δ*V*_m_) is induced across its membrane (Fig. 2). Induced TMV for a single spherical cell with a non-conductive plasma membrane can be determined analytically by solving Laplace equation in the spherical coordinate system, yielding the expression often referred to as the steady-state Schwan equation (Eq. 1) [16]. This induced TMV depends on (1) the amplitude of the local electric field (*E*), (2) the radius of the cell (*R*) (i.e., the same electric field induces larger TMV in larger cells), and (3) location on the membrane relative to the direction vector of the electric field (*θ*, is the angle between the specific location of the membrane and the direction vector of the electric field). The induced TMV is the highest on the poles of the cell facing electrodes. Although induced TMV can analytically be calculated for spheroids, it has to be determined either numerically or measured experimentally for realistic cell shapes [17, 18].

1 $${\mathbf{ - }}\Delta V_{\text{m}} = 1.5\,E\,R\cos \theta.$$

**Fig. 3:**
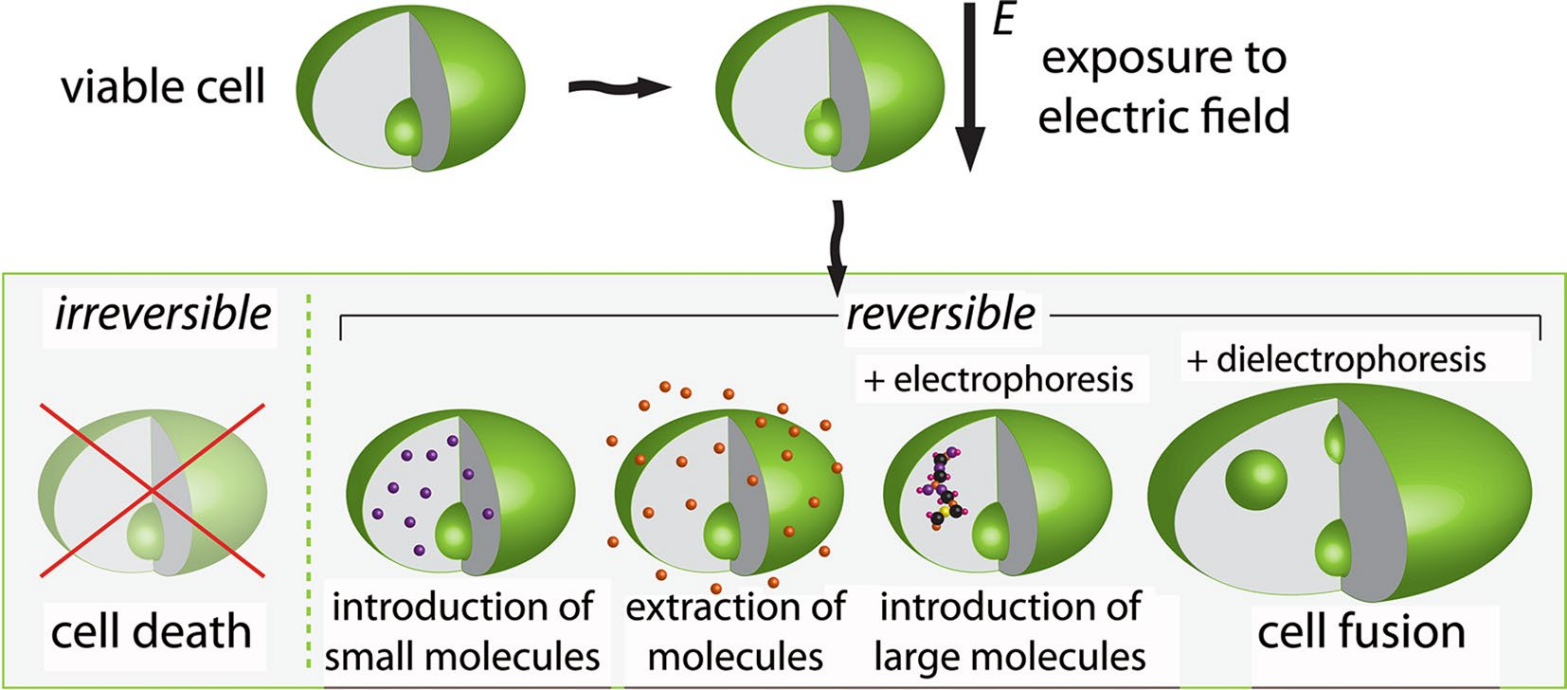
Schematic representation of processes with cells exposed to pulsed electric fields. The possible outcomes depend on the pulsed electric field protocol (amplitude, shape, number, and duration of pulses) and additional cell manipulation techniques, e.g., (di) electrophoresis. Exposure of cells to electric fields leads to increased cell membrane permeability due to electroporation. This phenomena can be used in biorefineries for killing of cells, fusing cells, extraction or introduction of small and large molecules into the cells Figure adapted from [30]

When the cell is exposed to sufficiently high electric field, the membrane becomes permeable for ions and molecules as large as pDNA, which otherwise are deprived of transmembrane transport. In this case, when this increase in membrane permeability is of a transient nature, and the cell survives and the membrane regains its selective permeability, electroporation is said to be reversible. If the cell dies, the electroporation is named irreversible. Both reversible and irreversible electroporation can be used in biorefinery applications (Fig. 3; Table 1).

**Table 1:** Reversible and irreversible electroporation pathways in biorefinery applications

Biorefinery application	Electroporation mode
Delivery of genes to improve feedstock phenotype and resistance	Reversible
Dehydration	Irreversible^a^
High-value products extraction	Irreversible
Biomass pretreatment	Irreversible^a^
Conversion into biofuels	Reversible and irreversible
Waste treatment	Irreversible

^a^The exact mechanism by which PEF affects lignocellulose biomass is not clear

Induced TMV even though considered relatively small (i.e., 0.2–1 V) is applied across only a short distance—the thickness of a cell membrane is in the order of 10 nm—which corresponds roughly to 1 MV/cm [13]. Recent molecular dynamics (MD) simulation results show that the electric field is non-homogeneous across the membrane [19]. This inhomogeneity can lead to even larger local electric fields. Such electric fields create pores in lipid bilayers, which depends on molecular composition of bilayer among others parameters [19, 20]. The working range of field strengths is usually 5–20 kV/cm for bacteria and archaea; 1–12 kV/cm for microalgae and yeasts; and 0.5–5 kV/cm for plants: [21, 22]. This roughly corresponds to the size of the cell—bacteria and archaea being the smallest, followed by yeast, and plant cells being the largest.

The MD simulations [19] confirm to a large extent a long time prevailing theory on formation of hydrophobic pores created due to membrane exposure to electric field. These hydrophobic pores are then converted into hydrophilic pores through which the transport of molecules occurs. This transport across electroporated membranes of normally impermeable molecules has been experimentally observed in bacteria, eukaryotic cells as well as in archaea [22]. Few artificial membranes have also been electroporated [23].

The TVM, induced by electric field is time-dependent and is expressed for a spheroid as described by [24] in the Eq. 2:

2 $$\Delta V_{\hbox{max} } = 1.5\,R\,E\,\exp \left( {{\mathbf{ - }}{t \mathord{\left/ {\vphantom {t {{{\tau }}_{\text{m}} }}} \right. \kern-0pt} {{{\tau }}_{\text{m}} }}} \right)$$

The associated charging time (τ_m_) is strongly dependent on the membrane capacitance, *C*_m_, internal (cytoplasmic), λ_int_, and external, λ_ext_, conductivities and on the microorganism size. The charging time of induced TMV decreases with an increase in the external solution conductivity.

3 $$\tau_{\text{m}} = \, C_{\text{m}} R \, ( 1/{{\lambda }}_{\text{int}} + 1/ 2 {{\lambda }}_{\text{ext}} )$$

The charging (τ_m_) time for *Escherichia coli* was found to be 17–50 μs in a 3 μS/cm (low conductivity) buffer ([25]. In the 0.2 mS/cm and 1 mS/cm buffers, τ_m_ was considerably reduced and was as short as 1 μs. For larger cells (plant cells), the steady-state TMV will not be reached when using short (a few μs) pulses as described in most experiments. Permeabilization will require higher field than when using longer (ms duration) pulses. The induced TMV in dense cell suspensions is smaller due to shielding of cells by neighbors [26]. The effect of pulse delivery frequency on the plant tissue permeabilization showed that for the onion tissue, more permeabilization occurs when pulses are delivered at 1 Hz than at 5000 Hz [27].

Although there are still gaps in understanding its basic molecular and cellular mechanisms, electroporation is successfully used in medicine [28, 29], biotechnology [22], and food processing [14, 30] and therefore, could enable industrial scale biomass processing (Fig. 3).

**Fig. 4:**
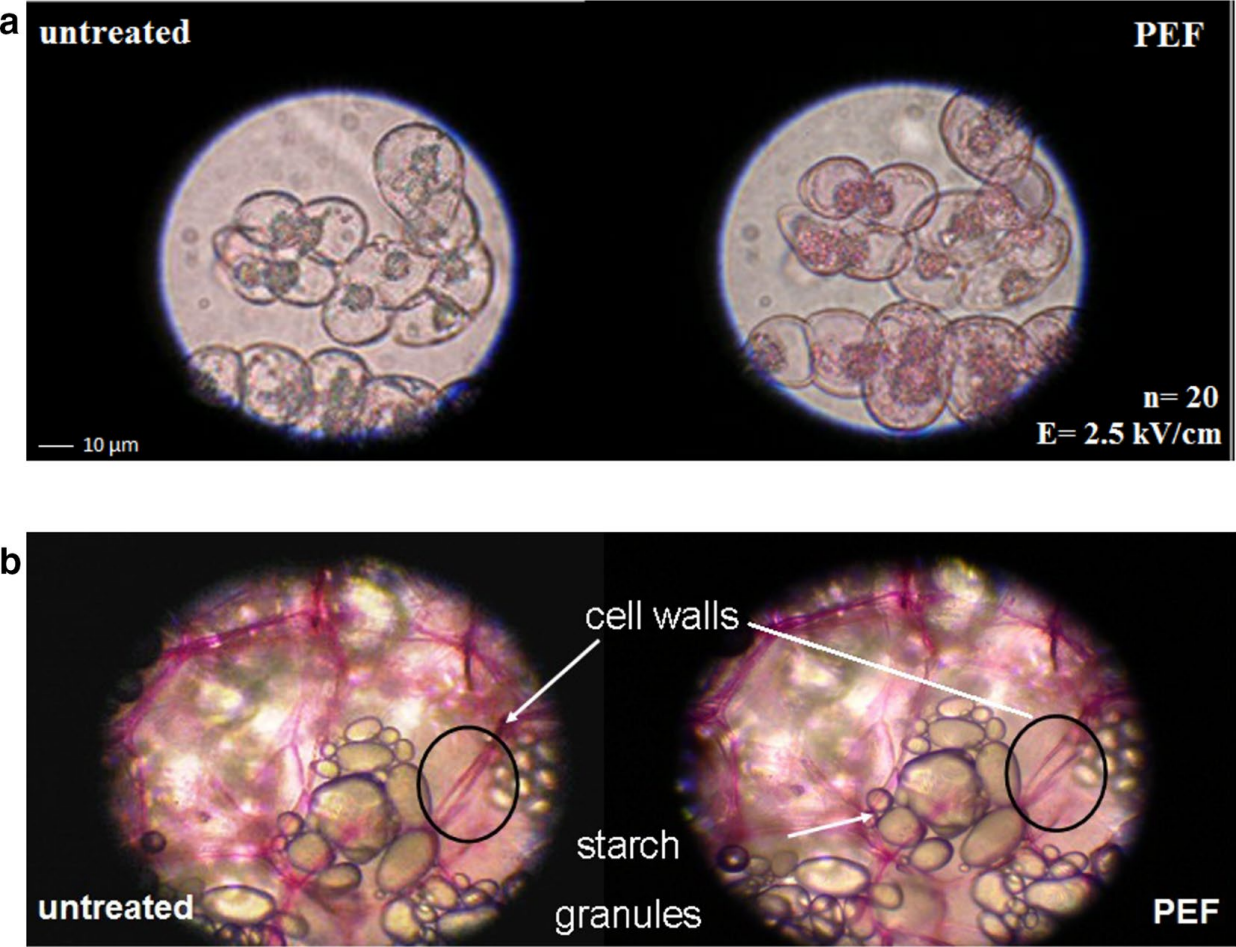
**a** Pulsed electric field effects on the *Nicotiana Tabacum* L. cv *bright yellow*-2 cells with cell wall stained with vital dye solution (Phenosafranine). The pulsed electric field protocol: *E* = 2. 5 kV/cm,* n* = 20, *f *(pulse frequency) = 2 Hz, exponential pulses with duration of 400 ms.** b** Pulsed electric fields effects on the extracellular matrix of potato. The pulsed electric field protocol: *E* = 5 kV/cm, *n* = 20, *f* (pulse frequency) = 2 Hz, exponential pulses with duration of 100 ms. Tissue staining was performed with ruthenium red 5 min after PEF treatment Figure adapted from [32]

A key effect of PEF applications in biomass processing today is the enhanced mass transport rate of extraction of different molecules [31], such as carbohydrates, lipids, pigments, phenols, lipids, and water [32] (Fig. 4). PEF can also be used to enhance the mass transport rate of small molecules, DNA and RNA penetration to the cells for genetic editing [31], (Fig. 2). In addition, PEF have been shown to affect the biomass structure, decrease lignin content an effect that could assist in deconstruction of the complex lignocellulose cell walls [33, 34] (Fig. 4). However, the exact mechanisms of PEF operation in this effect of lignin reduction are not clear. In summary, mass transport rate enhancing and structural changes in the biomass are fundamentals for the use of PEF technologies in biorefineries [35].

## Instruments for large-scale biomass processing

### Examples of devices for large-scale processing of biomass

From engineering point of view, high-mass-flow-rate PEF-processing of biomass with a given treatment energy requires large-volume treatment chambers and high pulse repetition frequencies. Large treatment volumes call for large electrode gaps, demanding for high pulse voltage amplitudes and large electrode areas, causing high current flow. In case of limited pulse voltage and current amplitude, mass flow rate can be enlarged by increasing pulse repetition frequency [36].

**Fig. 5:**
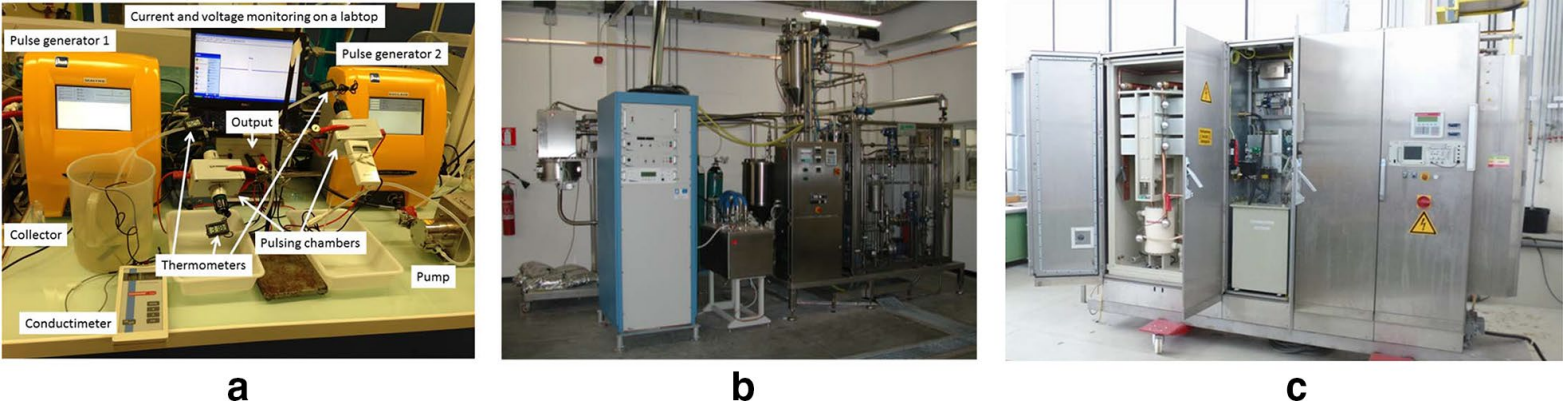
**a** Laboratory scale PEF system. Trains of two successive 2 kV 1(one) ms long pulses with opposite polarities are delivered at a 1 Hz frequency by 2 pulse generators on an array of pulsing chambers where a flow of cells is passing through at a 4 l/h rate. Measurement and control auxiliaries are indicated. **b** Pilot scale continuous flow (max flow rate 300 l/h) plant available at laboratory of ProdAl Scarl (University of Salerno, Italy) for PEF treatment of liquid biomass. It comprises, a DTI pulse generator (20 kV, 20 kW,* square *and bipolar pulses, 1–10 μs pulse width, 1–1000 Hz), four co-linear treatment chamber (0.32 cm inner diameter, 0.43 cm electrode gap), pump, heat exchanger, and storage tanks. **c** Pilot facility operative at KIT/IHM (Karlsruhe; Germany) for PEF treatment of dense cell suspensions at a mass flow of 400 l/h.* Rectangular-shaped *pulses of 65 kV and a duration of 1 µs are provided at a repetition frequency of 10 Hz, *left* section. The power supply is situated in the middle section. Measurement and control auxiliaries are installed in the *closed right section* Figure a adapted from [132]

**Fig. 6:**
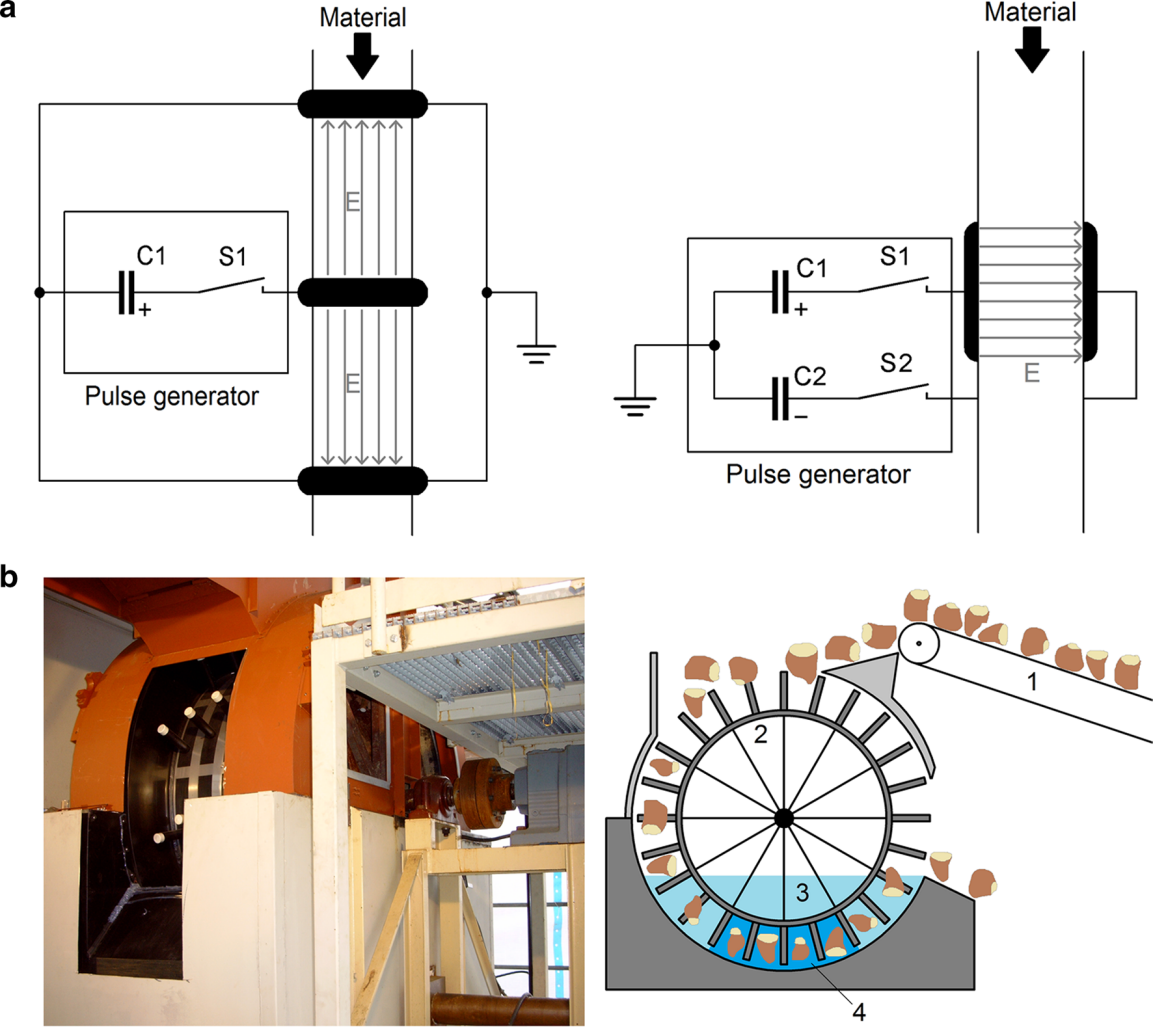
**a** PEF treatment reactor with collinear (*left panel*) and parallel plate (*right panel*) electrode arrangement each connected to a pulse generator. The material is transported through the electrodes and tubes passing two treatment areas between high-voltage- and ground electrodes. The electric field is oriented either in direction or counter direction of the material flow. In the parallel plate electrode arrangement, the orientation of the electric field is perpendicular to the material flow. The electrode system is fed symmetrically to ground potential by a pulse generator grounded at its center point. Hence, in a substantially homogeneous medium, ground potential is established in the center of the electrode system preventing leakage currents from flowing out of the electrode system toward inlet and outlet. **b** PEF treatment reactor for whole sugar beets developed by KIT/IHM (digital image, *left panel* and schematic representation, *right panel*): The sugar beets are transported by means of a conveyor belt *(1)*, to the top of a wheel equipped with electrically isolating rods *(2)*, when rotating the wheel the rods transport the sugar beets as a package through the PEF treatment reactor. The beets are immersed into water *(3)*, to establish an electric contact to the electrodes situated inside the PEF treatment area *(4)*

Fundamental and applied research on the PEF systems in the last four decades led to the development of technologies that enable large-scale biomass processing, required for the industrial scale biorefineries (for e.g., Fig. 5). To fulfill current requirements, large-scale PEF treatment devices comprise one or several pulse generators (for e.g., Fig. 6). They are electrically connected to an electrode system for continuous PEF treatment of a flow of material that is established by means of transporting the material through the device. For industrial applications, high reliability for continuous long-term operation and a competitive price for a return-of-invest within a short period of time is of importance. For providing high-voltage pulses, voltage adding e.g., according to the Marx principle is commonly applied [36]. Marx-type pulse generator enables voltage multiplication, while each stage component of the generator needs to be designed for the stage voltage only. Depending on the required pulse shape, a single stage can consist of a sole capacitor, a pulse forming network (LC-chain), or a transmission line [36]. A conventional Marx circuit, using single capacitors for energy storage, is designed to deliver an exponential-shaped, aperiodically or strongly damped periodically voltage waveform. An approximately rectangular pulse shape can be generated by means of a generator based on LC chains, either as single stage or also in Marx configuration [37], and in case of short pulses on the order of 1 µs and preferably less by cable pulse generators [38, 39]. Such a generator, however, requires matching of the load impedance to the generator.

The generators’ switching elements significantly determine generator performance. Spark gaps can switch voltages up to the MV range and currents exceeding several 10 kA, and are typical on-switches. Nevertheless, they only can handle repetition frequencies of several 10 Hz, whereas semiconductor switches are limited to switching-voltages of several kV or 10 kV, and currents on the order of 10 kA, as a rule, but, importantly, can switch off current and enable high pulse repetition frequencies in the kHz range.

Thus, a Marx circuit equipped with semiconductor switches capable of closing and opening a circuit and capacitors with a high capacitance can be used for generation of rectangular pulses and also stepwise arbitrary voltage shapes without exact matching of the load [40] and easily adjustable in pulse duration via semiconductor control electronics. In contrast, spark gap-switched pulse forming network generators also providing rectangular-shaped pulses, do not exhibit this flexibility in pulse duration, usually are not commercially applied for pulse durations exceeding 100 µs, but require less stages for realizing high output voltages and can handle higher currents compared to semiconductor-switched Marx topologies. However, if a large switching power in a small volume without necessity to open a circuit is required, currently spark gap-based switching solutions are still more competitive compared to semiconductor switches because of their lower price and for the possibility to design generators with higher stage voltages and a lower number of stage elements in consequence. Common stage voltage values of spark gap-switched Marx generators are between 50 and 100 kV. In the case of semiconductor-based systems, stage voltages are determined by the blocking voltage, i.e., 1.2–6 kV for standard devices.

To avoid complexity of semiconductor triggering of stacked systems, pulse transformer-based generator topologies exhibit advantages. In simplest implementation, a single semiconductor switch discharges a capacitor, charged to less than 10 kV, into the primary winding of a pulse transformer. Maximum output voltages and currents are limited by the transformer ratio and core saturation effects, respectively [41].

Transmission line generators, either spark gap-switched or semiconductor-switched, stacked, or single stage [42] did not enter large-scale PEF-processing of biomass so far, but might gain importance if future applications, e.g., stress response induction, require for pulses shorter than 1 µs and pulse rise times of less than 10 ns, which advantageously can be realized by these systems for voltage levels on the order of several 100 kV.

The design of the treatment chamber electrode system determines the way of grounding the pulse circuit. If the main direction of the electric field is parallel to the flow of material, grounding of one pole of the pulse circuit near the PEF treatment chamber is of advantage [43]. A collinear electrode design is an e.g., for such an arrangement [44]. In cross-linear chambers exhibiting a plate electrode design, the electric field is oriented in a 90° angle to the flow of material. For such field geometry, a ground-symmetric operation helps to prevent a leakage current from flowing out of the electroporation area [43]. The pulse circuit is grounded at the center of the pulse generator delivering a positive and negative voltage of half amplitude to the electrode system for PEF treatment. Hence, less effort for high-voltage insulation to ground is required and a device might be set up more compact [45]. Nevertheless, both configurations might be designed for the application of a roughly homogeneous electric field distribution.

For large-scale PEF treatment devices, the inductance of the pulse circuit may limit the amplitude of the pulse current at a desired pulse shape. Parallel configuration of several pulse generators overcomes such limitations. However, due to jitter of the switching moments of the generators energy oscillations between the generators may occur, if they are connected to one common pair of electrodes only. The use of single electrodes, which are aligned next to each other such that a damping resistor between adjacent electrodes is formed without influencing much the homogeneity of the electric field, prevents such energy oscillations. However, a low jitter of the switching moment is desirable. The jitter of spark gap switches can be reduced significantly by adding means for seed electron generation [46]. A corona discharge at the tip of a wire connected to the cathode of a spark gap emits ultra-violet light. The wire is placed next to both spark gap electrodes in such a way, that the light is able to generate seed electrons inside the spark gap without allowing the corona discharge to bridge the gap to the counter electrode of the spark gap. For more homogeneous radiation, a ring-like corona wire might be used instead. Additionally, a homogeneous-field profile of the spark gap electrodes lowers the jitter, because the volume with an electric field sufficiently large for streamer generation is increased and, hence, the probability for seed electrons to initiate a discharge [46]. Moreover, a homogeneous-field profile fosters homogeneous wear of the electrodes increasing their useful lifetime. Over-voltage triggering of the first stages’ spark gaps of Marx generators in parallel configuration enables triggering without additional wear [43]. An ignition electrode is omitted. An over-voltage is applied to each spark gap by means of one or two pulse generators replacing one or both charging coil between the first and second stage. Switching with low jitter is achieved by combining over-voltage triggering with a corona wire next to each spark gap.

The pulse generators might be supplied by the charging current of the Marx generator in order to simplify their integration into the circuit [47, 48]. The means for transporting the material through the PEF treatment device need to be adapted to the mechanical properties of the material. Crushed grapes for e.g., can be pumped. The use of two pumps enables a control of the pressure inside the PEF treatment zone in order to prevent the material from an electric breakdown when being treated at a high electric field level [45]. By means of a degassing valve, air might be removed from the material additionally increasing the electric breakdown strength of the material. Whole sugar beets, for e.g., as well as material with comparable mechanical properties might be transported through the PEF chamber by means of a wheel equipped with rods as a package [49]. So a constant velocity of the product all over the cross-section of the treatment area is guaranteed. Online measurement of the degree of cell disintegration enables an automated control of the device based on the material quality. The degree of cell tissue electroporation can be derived from impedance measurements of the material before and after PEF treatment [50, 51].

### Commercialization of industry level PEF technology

Currently, PEF technology is mostly widely used in the food industry. In the 1980s, the German equipment manufacturer Krupp has performed first attempts to commercialize the process, but at this time pulsed power switches have not shown sufficient performance and reliability [52, 53]. In the 1990s, in the US as well as Europe, consortia of food processors, equipment manufacturers, and universities have been formed to develop PEF applications and equipment [54]. In 1995 a continuous system was launched by PurePulse, a subsidiary of Maxwell Laboratories. In 2006, a first commercial installation for fruit juice preservation was achieved in the US but was stopped in 2008 due to technical and commercial limitations. The first commercial operation in Europe was achieved in 2009, with the installation of a 1500 l/h juice preservation line. In 2010, the first industrial system for processing of vegetables with a maximum capacity of 50 t/h followed. At present such PEF-treated food products are on market shelves in the Netherlands, Germany, and the UK, where PEF-processing equipment with a capacity of 1500–2000 and 5000–8000 l/h is used [55, 56]. An industrial system to enhance yield of cloudy apple juice is operated in a German fruit juice company in a 10 t/h scale [57]. Textural changes observed in potato, sugar beet, and carrot after a PEF treatment are caused by a loss of turgor pressure [58, 59]. As a result subsequent handling, pumping, or cutting processes may be facilitated. After treatment of potatoes with an energy input of 1–2 kJ/kg an improved cutting is observed, causing less fracture, and a smoother cut surface after industrial hydrojet cutting. Due to tissue softening, less product breakage occurs in the following production stages. The process is currently used with a number of 40 industrial installations to replace conventional pre-heating of potatoes (60 °C, 30 min) in French Fries production [56]. In Table 2, we provide an overview of industrial scale pulse generators, as far as data are available. These developments of commercial systems for the food industry will enable the commercial use of the PEF systems for other biorefinery application such as chemical production and biofuels.

**Table 2:** Industrial PEF systems

Manufacturer	Power (kW)	Max voltage (kV)	Max current (A)	Treated material
Diversified Technologies (USA)	1–150	40	300	Liquids
ELEA (Germany)	5–80	40	200–5000	Liquids and solids
Energy pulse systems (Portugal)	3.5	10	150	Liquids
KEA TEC (Germany)	25	300	7000	Liquids and solids
Maxwell pulse (USA)	NA	40	NA	NA
Pure pulse (The Netherlands)	16–30	40	NA	Liquids
ScandiNova (Sweden)	0.4–90	10–450	20–1000	NA
SteriBeam (Germany)	3	20–30	NA	Liquids and solids
Pulsemaster (The Netherlands)	80	NA	NA	Liquids and solids

*NA* not available

### Electrochemical reactions due to pulsed electric field application

Importantly, when PEF are applied to biomass (or any electrolyte solution) placed in direct contact with the metal electrodes of the treatment chamber, besides electroporation of the biological cell membrane, i.e., which makes it permeable for molecules otherwise deprived of any transport mechanisms, a variety of electrochemical reactions occur at the electrode–solution interfaces [60, 61]. These reactions are undesired and their extent should be minimized since they may cause problems in different fields.

For e.g., the electrode reactions can cause evolution of gas (H_2_, O_2_), produce toxic chemicals (mostly H_2_O_2_, HCl, HClO), electrolysis of water, and changes to the chemical properties (pH, electrical conductivity) of the processed fluid in the vicinity of the electrode surfaces and dissolution of the electrode’s material [60, 62, 63]. In addition, reaction products that are formed can react in the bulk with other compounds leading to the formation of toxic compounds or degradation of biomaterials even after PEF treatment has been completed. Electrochemical reactions may also lead to fouling that during extended processing time they can cause several problems such as local electric field distortion, arcing, contamination of the treated material, and in some case, cause the flow of the fluid product to stop. Finally, corrosion can cause serious damages to the electrodes, whose surface roughness can increase as a consequence of the metal release. This, in turn, can cause local electric field distortion and arching, drastically limiting the life time of the electrodes to few hours of operation only [64, 65].

The extent of all the above undesired effects related to the electrode reactions depend on many factors such as chamber design and electrode’s material, electrical parameters such as pulse shape, peak voltage, total specific energy input, polarity, and pulse duration as well as the composition and chemical–physical properties of the treated products [66–68].

Although a number of studies have shown that, the extent of the electrochemical reactions may be limited by either using electrode materials featuring higher resistance to electrochemical reactions such as titanium, platinized-titanium or conductive polymers [65], or by using bipolar pulses [66], dissolution of electrode materials and other electrochemical reactions are largely unavoidable in the long term.

Numerical simulation has been recently applied as a valuable tool to predict the occurrence of the electrochemical phenomena at the electrode-liquid interface of a treatment chamber and to optimize the process with respect to the chamber design, electrode’s material, product pureness and composition, and the range of operating conditions [69].

## PEF treatments for biomass feedstock development

### PEF for gene delivery to improve plant biomass feedstocks

For plant cell transformation, often required for genetic modification of the biomass feedstock, the cell wall is often considered as a barrier to DNA transfer, which is only overcome by rupture or wall-degrading enzymes (protoplast formation) [70, 71]. Using PEF for plant cell transfection was mostly described after protoplast formation [72]. Indeed cell plasmolysis before PEF is an efficient approach to DNA delivery into intact plant cells [73]. Using PEF, transient expression (β-glucuronidase (GUS) and chloramphenicol acetyltransferase (CAT)) and stable expression (phosphinothricin acetyltransferase) of exogenous genes were obtained in intact black Mexican sweet maize cells a crop difficult to transform [73]. In this work, one single 15 × 10^3^ µs long pulse of 750 V/cm was applied, leading to a 100 % increase in transient GUS expression in intact maize cells, while preserving the cell viability, by adding 10 mM ascorbate just after PEF.

### PEF for gene delivery to improve algal biomass feedstock

Stable transformants of both wall-less and walled strains of the microalga *Chlamydomonas reinhardtii*, *Chlorella ellipsoidea*, and *Dunaliella salina* have been obtained using PEF [74–76]. Temperature, osmolality, electric conditions, field strength (kV/cm), time of discharge, and DNA concentrations have to be carefully optimized to obtain high transformation efficiencies. In [77] it was reported that high efficiency of transformation was achieved in *Chlamydomonas* to 2 × 10^5^ transformants per μg of DNA, about two orders of magnitude higher than that obtained with the standard glass beads method to introduce exogenous DNA. More recently stable gene transfer by PEF was established in other eukaryotic microalgae, including *Nannochloropsis* sp. [78, 79] *Scenedesmus obliquus* [80], *Chlorella vulgaris* [81], and *Phaeodactylum tricornutum* [82, 83].

A common rule for the published protocols was that cells were grown phototropically and harvested in the exponential phase of growth. No pretreatment was described. The cultures were washed in a pulsing buffer that was optimized for each trial but in most cases was poor in salts to reduce the conductivity (longer pulse decay, limited Joule heating). A high cell density was used with several μg of pDNA (that could be linearized). Field strengths ranged from 2.5 to 11 kV/cm with decay time from 3 × 10^3^ to 26 × 10^3^ µs. Most trials were performed with a capacitor discharge system except in a recent paper on *P. tricornutum*, where cells in the log phase of growth were resuspended in an hypotonic buffer and submitted to PEF (several pulses 1.5 kV/cm, 5 × 10^3^ µs followed by a train of 40 V/cm, 50 × 10^3^ µs) [83].

The lack of pretreatment to weaken the wall in algae was the opposite to what was routinely used on yeasts. Pretreatment of the *Saccharomyces cerevisiae* yeast cells in the early phase of exponential growth with dithiothreitol increased transformation efficiency [84]. Transformation efficiencies of 10^7^ transformants/µg of plasmid DNA were obtained with a square wave electric pulse of 2.7 kV/cm with 15 × 10^3^ µs pulse length. Even small quantities of DNA (100 pg) can be used to transform 10^8^ cells. Important parameters are the pulse field strength, to which cells are exposed, and duration. The method has been successfully applied to various strains of *Saccharomyces cerevisiae* as well as to other types of yeast [84–86].

## Biomass dehydration with pulsed electric fields

Dehydration is one the most energy intensive processes in the biomass treatment. PEF enable energy-efficient dehydration. For example, in the course of the production of sugar from sugar beets, PEF-assisted drying of cossettes increases, in combination with alkaline extraction of the sugar, the dry matter content of the cossettes after pressing from 35 to 40 % [87, 88]. In a conventional sugar-production process, lime milk is used for purging the juice and, hence, added to the juice after extraction. For alkaline extraction, the lime milk is added to the cossettes before diffusion [88] or between two pressings [87]. It strengthens the cell walls, so during pressing water may better drain out of the material. The lower water content results in lower evaporation energy during the subsequent drying stage for the cossettes. PEF-assisted drying is not only more efficient due to the decreased water content after pressing. PEF-treated material exhibits a better diffusion of water and vapor, thus less time is required for the drying process. The decrease of humidity with the time is for PEF-treated material much steeper than for untreated material. The time required for the drying process of energy crop in an oven could be reduced by a factor of 2–3 compared to non-PEF-treated material [89]. For energy-efficient dehydration of green rye and food crops, an electrified press has been set up [51, 90]. Such a press enables the application of high-voltage pulses and mechanical force simultaneously. No extra water needs to be added, because the electric contact to the electrodes is established by means of the juice, which is initially pressed out of the material. When using a stamping press, additional pulse application may increase the yield of juice approximately by a factor of two compared to pressing only.

## Biorefinery processing enabled by pulsed electric fields

### Phytochemicals extractions from biomass waste

The increasing production of agri-food waste or by-products is a matter of concern by the industry mainly due to their environmental, economic, and social impact. However, nowadays, the approach toward these material is changing since they are considered as a cheap source of valuable components, such as natural colorants (anthocyanins, carotenoids, betanin, etc.) or nutraceuticals (polyphenols) that can be recovered and used as functional additives in different food and pharmaceutical products [91]. The extraction of these high added-value compounds, which are generally confined inside the plant cells, is mainly limited by the resistance to mass transfer through the cell membranes (cytoplasmic and tonoplast membranes) and the cell wall [92, 93]. For this reason, in order to improve the extraction yield, traditional extraction techniques are generally preceded by raw material pretreatments, namely grinding, heating, addition of chemicals/enzymes, which, however, have a negative impact on the quality of the extracts [93, 94]. Moreover, conventional extraction methods are time-consuming, use large amounts of solvents, high extraction temperatures, and may require the product to be dried. Consequently, demand is increasing for green and sustainable extraction techniques that improve yield, shorten the extraction time, and reduce the use of organic solvents [95].

PEF have been demonstrated to be a promising mild and more efficient physical method alternative to conventional cell disintegration techniques [94]. An enhancement in the extraction yield of phenolic compounds, flavonoids, anthocyanins, carotenoids from agricultural and food processing by-products of artichoke [96], blueberry [97], grapes [98] and grape seeds [99], flaxseed hulls [100], orange peel [101], and tomato [102] has been reported when PEF treatment is used in combination with either mechanical pressing or extraction with solvents. In particular, when PEF is applied in combination with a solvent extraction, the latter should be selected taking into account several factors, such as the solubility of the compounds of interest, its ability to penetrate or diffuse into the solid matrix, as well as its influence on the electrical conductivity of the treated biomass, which could affect the performance of the PEF treatment itself. Interestingly, in [96], PEF treatment was applied as a pretreatment stage of electroporation of cellular membranes in the extraction process of valuable components, such as polyphenols, from involucral bracts of artichokes. The vegetable by-product was exposed to two different PEF treatments at varying electric field intensities and total specific energy inputs (0.75 kV/cm and 0.5 kJ/kg; 1.5 kV/cm and 5 kJ/kg) at fixed pulsed width (10 μs) and pulse repetition frequency (10 Hz). The treatment obtained was evaluated by measuring electrical impedance of the tissue before and after treatment. Extraction process was performed by liquid solvent extraction in water for 24 h. Results showed that increasing the intensity of the PEF treatment increased the permeabilization of bracts tissues leading to a cell disintegration index (*Z*_p_) equal to 0.5 for the first protocol and 0.9 for the second protocol. In agreement with these results, the application of the PEF treatment accelerated the extraction rate of polyphenolic content as compared with the control (untreated sample). The final extraction yields increase of 27 % for the first protocol and 150 % for the second protocol.

**Fig. 7:**
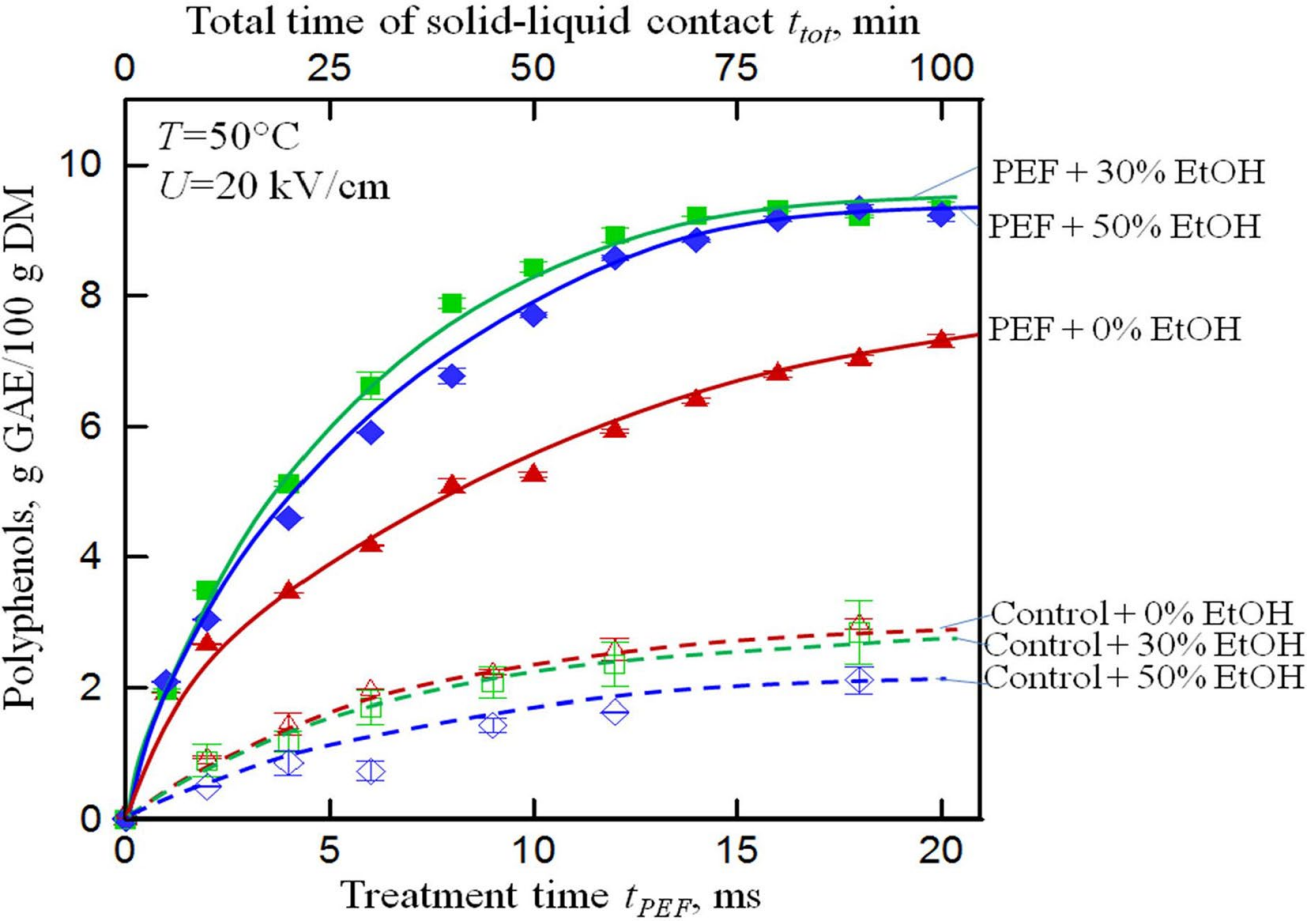
Effect PEF treatment and ethanol solvent parameters on extraction of polyphenols from grape seeds. Coupling of PEF to ethanol solvents at optimum concentration increased the total yield of polyphenols extraction Figure adapted from [99]

Grape by-products (pomace, peels, seeds, and vine shoots) are very rich in bioactive compounds and especially in polyphenols (anthocyanins, catechins, flavonol glycosides, phenolic acids, etc.). The effects of PEF-assisted recovery of total soluble matter and polyphenols from grape skins, pomace, peel, and seeds were studied in aqueous media and water–ethanol solutions at different temperatures within 20–60 °C [99, 103]. The PEF treatment (8–20 kV/cm, 2 × 10^3^–20 × 10^3^ µs) permitted considerable increase of the yield of extractives especially at higher PEF intensities (Fig. 7).

PEF pretreatment was also applied to enhance extraction of soluble solutes from fennel (Foeniculum vulgare) [104]. Fennel is a medicinal and aromatic herb and its extract contains valuable antioxidants. Optimal extraction with the maximal juice yield (98 %) was obtained at *E* = 350 V/cm. Effects of PEF treatment on extractability of phenolics from fresh spearmint leaves were studied [105]. The highest disintegration was obtained at a PEF intensity of E = 3 kV/cm with a specific energy input of ≈4 kJ/kg.

Extraction of total polyphenols and flavonoids from orange peel using pressing and PEF (*E* = 1–7 kV/cm) was studied [101]. The total polyphenols extraction yield and antioxidant activity of the extract were increased up to 159 and 192 %, respectively, if PEF was applied after pressurization at 5 bars. The PEF-assisted extraction (*E* = 13.3 kV/cm) from mango peels was studied at different temperatures (20–60 °C) and pH (2.5–11) [106]. The application of the two-step procedure including PEF-assisted and supplementary aqueous extraction at 50 °C and pH = 6 allowed considerable enhancement of the polyphenols yield (+400 %).

### Phytochemical extractions from lignocellulosic biomass

Lignocellulosic biomass from terrestrial plants, energy crops, and crop residues has good potential for biofuel or biogas production and recovery of highly added compounds, e.g., phenolic compounds. PEF-assisted extraction of polyphenols from Norway spruce *Picea abies* at 20 kV/cm and pH 12 was studied [107]. The polyphenols extraction yield was increased ≈10 times when compared to untreated samples. It was concluded that the application of PEF-assisted extraction treatment seems to be a good alternative to the high temperature extraction process. PEF-assisted extraction of polyphenols from wood barks was also investigated [108, 109]. Data demonstrated that the polyphenols extraction by PEF (20 kV/cm and 2 × 10^3^ µs) application is increased. The final extraction yields obtained with PEF (4.94 ± 0.42 g GAE/100 g DM) were close to that obtained for the grinded product (6.04 ± 0.29 g GAE/100 g DM). The reported energy consumption of PEF (3.2 kJ/g) was, however, lower than that of grinding (8.75 kJ/g).

PEF treatment (1.25–2.5 kV/cm) was applied to alfalfa [110]. Alfalfa contains crude protein (15–20 %) of high nutritional quality, vitamins, and different kinds of minerals. Significant tissue damage leading to the increase of juice yield (by 38 %) and dry matter during mechanical expression at 2 and 4 MPa was observed. Protein and mineral contents in the treated samples were also significantly increased.

Rapeseed stem is a green biomass feedstock generated from the rapeseed oil production. Experiments conducted under optimal conditions (*E* = 8 kV/cm, *t*_PEF_ = 2 × 10^3^ µs, *P* = 10 bar) permitted to increase the juice expressed yield from rapeseed stem from 34 to 81 %. Significant increases in total polyphenols content (0.48 vs 0.10 g GAE/100 g DW) and total proteins content (0.14 vs 0.07 g BSA/100 g DW) were observed after PEF pretreatment. The recovered press cake was well dehydrated with an increase of dry matter content from 8.8 to 53.0 % [111]. Olive kernel is rich source of both antioxidants (vitamin E, polyphenols, chlorophylls, carotenoids) and proteins with great potential of utilization in new food products, food additives, and nutraceuticals. The effective extraction with PEF-assisted methods required a relatively low energy input (60–80 kJ/kg) to obtain the polyphenols yield of 146 mgGAE/l [112].

### Pulsed electric field systems for energy-efficient microalgae fractionation

Microalgae are known to be the most productive biomass feedstock nowadays. Cultivation in closed photo-bioreactor (PBR) systems on barren lands avoids competition with food production, saves water and nutrition resources, and can provide production rates of 40–80 t ha^−1^ a^−1^ [113], which is 2–5 times higher as obtained for agricultural produced biomass [114]. The content of valuable components to be possibly marketed, i.e., lipids, proteins, carbohydrates, antioxidants, and vitamins, covers the entire biomass. Unfortunately, biomass density after cultivation is below 10 g_dw_/l [115] and most products of high interest are confined intracellular, protected by a rigid cell wall and the cell's plasma membrane.

The energy content of microalgae (chemically stored energy) depends on lipid content and ranges between 20.6 and 26.7 MJ/kg_dw_ [116]. The lower value was obtained from microalgae with 20 % of accumulated lipids. An energy demand for cultivation, 10 MJ/kg_dw_, is reported for flat panel photobioreactors [117]. Concentration of microalgae biomass to a density of 100–200 g_dw_/l, required for efficient further processing, also requires at least 3.6 MJ/kg_dw_ for centrifugation [118, 119]. Conventional cell disruption for efficient component extraction additionally consumes energy on the same order of magnitude under optimum treatment conditions [120]. This illustrates that in particular for an energetic use of microalgae all additional energy-consuming downstream processing steps, i.e., drying, washing, have to be avoided. Moreover, it is commonly agreed, that besides lipids for biofuels additional components concurrently have to be valorized for bulk chemicals, food, and feed to strive for economic viability [121, 122].

PEF treatment, involving plasma membrane electroporation as basic biophysical process, was shown to be an efficient wet-route processing technique exhibiting fractionating properties [123]. When treating microalgae suspensions of *A. protothecoides*, pre-concentrated to 100 g_dw_/l, with rectangular 1 µs pulses of an electric field strength of 34 kV/cm, 15 % of the total biomass could be released into the extracellular medium right after PEF treatment [124]. This water-soluble fraction contained salts, sugars, amino acids, and soluble proteins. Due to their size of ~1 µm and larger, intracellular oil bodies could not pass cell wall and permeabilized membrane and remained intracellular. After separation of the water-soluble fraction, lipids were extracted with Ethanol from the residual, lipid-rich biomass fraction. The lipid yield from the PEF-treated residual fraction was 3–4 times higher, compared to the untreated sample, recovering more than 80 % of the stored lipids on average [125]. PEF treatment was performed without preceding washing steps at an initial conductivity of 1 mS/cm which represents the conductivity of the cultivation medium at the time of harvesting. The required treatment energy was 150 kJ per liter of treated suspension and 1.5 MJ/kg_dw_, respectively. Furthermore, it could be shown that the extraction efficiency did not decrease at higher biomass densities [124]. Consequently, microalgae suspension of 200 g_dw_/l requires a specific treatment energy of 0.75 MJ/kg_dw_, which is considerably lower compared to conventional processing. Moreover, PEF treatment does not produce cell debris, which facilitates subsequent separation processes.

These merits of PEF processing satisfy the demand for a low energy-consuming technology for cascade valorization of microalgae biomass for an energetic use of the lipid-rich fraction. PEF-assisted fractionating component recovery allows for compensating the comparatively high energy demand for cultivation by simultaneous valorization of higher-value water-soluble products and might also open new processing-route pathways for microalgae exhibiting a high net-energy-balance use in energy application [22].

Microalgae are also attractive for the production of molecules including natural and recombinant proteins [126, 127]. However, extraction of proteins is hindered by the cell wall barrier. Namely, only a slow excretion was present on the microalgae having a rigid cell wall and therefore, disrupting the rigid cell wall of *C. vulgaris* was required to obtain protein release after extraction [128].

**Fig. 8:**
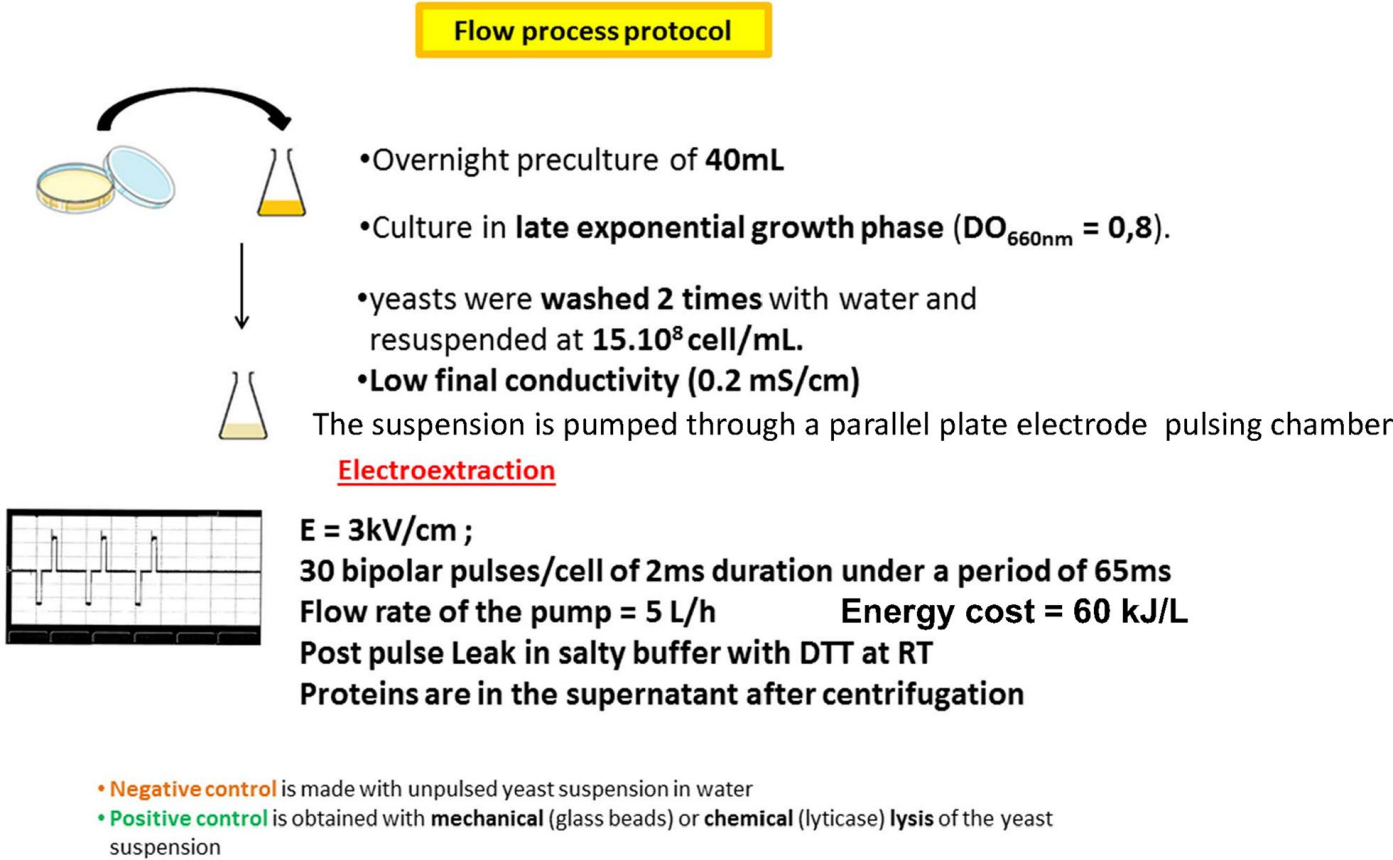
Flow process protocol for protein electroextraction from yeasts. In the lab scale pilot configuration, the volume of the pulsing chamber is set to 1.5 ml. Due to the low solution conductance, the current is only 1.2 A for the voltage of 1.8 kV needed to get the field strength of 3 kV/cm. Under the pulsing parameters (30 pulses per second with 2 × 10^3^ µs duration), the average power is 130 kW/l. A 26 °C temperature increase is associated to the flow PEF but cooling after the treatment is fast. This explains why there is no loss of enzymatic activity in the extracted proteins Figure adapted from [129, 131]

PEF were described as one of the most promising approaches for molecules extraction from microalgae. Long pulses (10^3^ µs long) appeared to electroporate the plasma membrane increasing its permeability and to induce structural changes in the wall. As a final consequence, a slow release of soluble cytoplasmic proteins was obtained without formation of debris, which is usually hindering downstream purification. PEF conditions can be adjusted in such a way to leave the vacuole intact, to prevent the release of proteolytic enzymes. The proof of concept of the flow process protocol to treat industrially significant volumes was previously reported [129] (Fig. 8). The optimum number of pulses was delivered on each algae cell during its residency in the pulsing chambers. One obvious physical problem was that due to the Joule effect, the temperature increased and there was a need of an array of pulsing chambers. Long square-wave pulses not an accumulation of short pulses were proved to be needed to obtain cytoplasmic soluble protein extraction [129, 130]. The use of long electric pulses was associated to a technical drawback: electrochemical reactions are occurring at the surface of the electrodes. This was largely prevented by delivering trains of pulses of alternating polarities with a short pause (about 10^4^ µs or less) between each (Fig. 8).

Electric fields of 4.5–3 kV/cm were efficient on fresh water-grown *C. vulgaris* and *H. pluvialis,* while 6 kV/cm was needed for salty water *N. salina* due to its smaller size [131]. Protein extraction was assayed by the coomassie blue assay and SDS-PAGE. The bands in the PEF samples appeared between 35 and 170 kDa. The bands of the controls were present with a higher intensity for the pulsed samples. Several new proteins appeared after PEF extraction. Importantly, no smear of the bands was observed after the overnight incubation supporting the lack of protease activities. The extraction increased with the increase in the electric field strength applied.

Pulse duration was clearly a leading factor to obtain protein extraction from walled species [131, 132]. A single pulse duration of 2 × 10^3^ µs was efficient. Microsecond long pulses were not inducing the protein release even if the cumulated application time lasted several ms [130]. The amount of released proteins was increased with the number of successive pulses and a highly efficient protein extraction was obtained with one cycle of 15 bipolar pulses. Protein leakage from microalgae was slow. A massive leakage was obtained during the first 30 min following the electro-treatment. A more complete extraction was obtained after an overnight incubation at room temperature. The pathways for protein leakage could not be detected as no ultrastructural damage was observed. Additional process parameters such as lysing buffer strength, medium pH, and PEF-processing temperature have been also shown to play a role in the extraction from microalgae proteins yields [133–135].

### Molecules extraction from bacteria and yeast

Walled microorganisms are cell factories, a basic unit for biorefineries. Heterologous protein production is routinely obtained in the Gram-negative bacterium *E. coli*. It can grow rapidly and at high density at low production costs. Its genetics is well-characterized and a large number of cloning vectors and mutant host strains are available [44, 125]. Yeasts (*S. cerevisiae, Kluyveromyces, Pichia, and Hansenula*) are widely used for industrial production of homologous proteins. Nowadays, they are recognized as a suitable host for industrial production of recombinant proteins with high added-values [136, 137].

Cytosolic protein secretion across the cell wall is impossible or of low efficiency. Thus, the newly synthesized homologous and heterologous proteins remain accumulated in the cell cytoplasm. Mechanical disintegration and chemical extraction are needed for protein extraction. PEF treatment was described as one of the most promising approaches for protein extraction. Long pulses (10^3^ µs long) appeared to electroporate the plasma membrane thus increasing its permeability and to induce structural changes in the wall. As a final consequence, a slow release of soluble cytoplasmic proteins was obtained without formation of debris, a bottleneck of downstream processing, also PEF conditions can be adjusted in such a way to leave the vacuole intact, to prevent the release of proteolytic enzymes.

PEF-assisted extraction from *E. coli* was strongly dependent on the growth phase of the pulsed microorganisms. The PEF-induced release of enzymes from cells in late exponential growth phase was only 50 % of what was obtained in the middle exponential phase. When the cells were in stationary growth phase, the electric field was completely inefficient. Cells were treated with 15 pulses, 4 Hz, 0.5 and 1 × 10^3^ µs duration, followed by a post-pulse incubation at 30 °C. Maximal release was obtained for glyceraldehyde-3-phosphate dehydrogenase (GAPDH) at electric field intensity of 7 kV/cm and pulse duration of 0.5 × 10^3^ µs. The upper limits in the field strength were detected by formation of precipitates and a decrease of GAPDH activity tested. This may be due to an increased joule heating during electric treatment.

The common buffer for protein extraction with *E. coli* is Tris buffer pH = 8–8.5 with EDTA and DTT as additives. Not only the plasma membrane was affected, but the wall organization was altered as an increase in sensitivity to wall lytic enzymes (lysozyme) at low concentration was obtained. PEF was used for cytoplasmic proteins extracted from yeast [138–140]. Protein release from the cells is a slow process occurring during the incubation in the specific buffer within several hours. Purification was then performed by classical methods for soluble proteins [141].

The extraction yield depends on the field strength, pulse duration, and the number of pulses delivered. Optimization is cell strain-dependent. DTT brings a significant increase in extraction due to the effect on the wall. Extraction from *S. cerevisiae* was obtained with 15 pulses of 2 × 10^3^ µs at 6 Hz. Maximal yield for GAPDH (145 kDa) (85 %) was obtained at 3.2 kV/cm where all cells were permeabilized which coincided with plasma membrane permeabilization as determined by Propidium Iodide assay. The maximal release of 3-phosphoglycerate kinase (PGK, 45 kDa) and hexokinase (HK, 100 kDa) was obtained during the same time. Periplasmic enzymes such as invertase could be extracted [142, 143]. The specific activity of three extracted enzymes (GAPDH, PGK, and HK) was about two times higher than that was obtained in cell extracts, from either after enzymatic lysis or mechanical grinding. PEF-treated cells incubated in isotonic medium showed Lucifer Yellow (LY) fluorescence only in the cytoplasm, the vacuole remaining unstained—showing that the vacuolar membrane was intact, preventing the release of proteases. Importantly, an increase of the PEF intensity above the optimal value led to a decrease of enzyme activity. An increase of intensity from 2.7 to 3 kV/cm resulted in about 90 % decrease of extracted GAPDH activity.

The incubation of pulsed cells at 30 °C rather than at room temperature did not affect the efficiency of extraction. This is a positive advantage in running costs. The presence of glycerol and DTT in the post-PEF incubation medium contributed to higher GAPDH (about 15 %) and PGK (about 20 %) activities from PEF-treated cells but did not influence hexokinase activity. No major structural alterations of the cell wall were observed by electron microscopy after the PEF treatment.

### PEF treatment of substrate for enhancement of biogas yield in anaerobic digestion

Energy and cost-effective biogas production is essential for the future renewable energy-based electricity production. The biogas process depends on bio-availability of molecules bound within cells, clumps, and other conglomerates. The rate limiting step is the hydrolysis where raw substrates of high molecular weight, such as proteins, carbohydrates, and triglycerides are cracked [144]. As a result, dimers and monomers, such as amino acids, fatty acids, and sugar mono- and dimers, are available for subsequent digestion. A critical delay during hydrolysis is the slow release of digestible material from cells or supercellular structures. Breaking clumps and large crop debris by milling has been shown to be very effective. Other methods for pre-processing like heating, ultrasound, microwave treatment, or shockwaves are under investigation and are becoming commercially available [145].

A very effective method for disintegration of cells and cell organelles is the application of PEF [146]. PEF treatment accelerates the hydrolysis step and is also effective for a higher level of digestion, especially in the case of waste management. The optimization of PEF protocols focusses on economic measures like the additional gas yield with respect to the applied energy, the reduction in hydraulic retention time, and the final content of biosolids after digestion. The PEF treatment outcome exhibits a non-linearity dependence between PEF parameters and improvement biogas yield. Depending on the substrate and total time of electrical treatment (pulse number × pulse duration), a threshold of the electric field between 10 kV/cm and 30 kV/cm was reported. Even for the same applied energy, less but more intense pulses can be more efficient than more pulses with lower field strength [147].

The most pronounced effect of the electric field on the raw substrate is the increase of bio-availability of nutrition by cracking of cell structures. This yields faster digestion and therefore a shorter retention time. The treatment of re-circulation, i.e., substrate pumped out of the digester through the pulse chamber and back into the tank, does not primarily aim to enhance the cracking of cells but aims to improve the efficiency of the anaerobic digestion process by the microbial community as it is expected from diverse field effects on several cell lines [148, 149]. The optimization is difficult because of the many variables yielding a highly variable outcome. Essentially for each single digester and every substrate, an adjustment of the PEF treatment protocol is required. The economic measure for optimization is the specific biogas yield with respect to total organic mass which in turn decreases the content of biosolids after digestion.

## Conclusions and future directions on the electroporation-based technologies for biorefineries

PEF processes have already shown very exciting results pushing forward multiple aspects of biorefineries, ranging from feedstock development, through dehydration and products extractions to waste treatment (Table 3 shows several examples of PEF use in different stages of biorefineries). PEF treatment is non-thermal and less energy-consuming compared to the conventional thermal extraction and dehydration operations, and permits to better valorize plant cell compounds.

**Table 3:** Examples of PEF application in biorefineries

Biorefinery application	Examples	PEF parameters	Achieved effects
Delivery of genes to improve feedstock phenotype and resistance	Plants: Mexican sweet maize	750 V/cm, 15 × 10^3^ µs pulse duration, single pulse	Transient expression of GUS and CAT, and stable expression of phosphinothricin acetyltransferase [73]
	Algae (strains development): *Chlamydomonas*	~1900 V/cm, single pulse of exponential shape with 10 µF capacitor discharge	2 × 10^5^ transformants per µg of DNA [77]
Dehydration	Sugar beet cossettes	3–5 kV/cm, 1.6 µs pulse duration, 40–80 pulses [88] 600 V/cm, 100 µs pulse duration, 100 pulses, 2.76 ± 0.16 Wt/kg [87]	PEF reduced the force required for a beet slicing from 16 to 8 N, reducing the total process energy requirement, and costs on changing the blades. In addition, combination with lime reduced the extraction process temperature from 72 to 60 °C with the same extraction efficiency [88] Lime improved the drying efficiency: 40 % dry matter content of the pulp was archived with less energy invested in evaporation than in untreated samples [87, 88]
	Green rye	3.5 kV/cm, 1.5 µs pulse duration, 80 pulses	8 % reduction in relative humidly after PEF + pressing with extrusion press. 100 min reduction time in drying under 105 °C in comparison with untreated controls [51]
	Grass, maize, and lucerne drying	7 kV/cm, 1.5 µs pulse duration, 40–80 pulses	>50 % energy saving in comparison with traditional methods [89]
High-value products extraction from biomass waste	Polyphenol extraction from involucral bracts of artichokes	5 kV/cm, 10 µs pulse duration, 100 pulses, 5 kJ/kg, pulse repetition frequency 10 Hz	Almost totally destroyed membranes according to disintegration index (*Z*_p_ = 0.9) extraction solvent: water. Extraction yield of polyphenols increased by 150 % in comparison with untreated samples. [96]
	Polyphenol extraction from grape by-products (pomace, peels, seeds, and vine shoots)	20 kV/cm, 10 µs pulse duration, 2000 pulses, pulse repetition frequency 0.33 Hz	20 g GAE/gDW extraction yields [99, 103]
	Total polyphenols and flavonoids (naringin and hesperidin) extraction from orange peel	7 kV/cm, pulse duration 3 µs, 20 pulses, pulse repetition frequency 1 Hz 5 kV/cm 3 µs, 20 pulses, pulse repetition frequency 1 Hz	Increased the total polyphenol extraction yield by 159 % [101] 3.1 mg/100 g yields of naringin and hesperidin [101]
Lignocellulose biomass pretreatment	Wood chip	10 kV/cm, 100 µs pulse duration, 2000 pulses, pulse repetition frequency 3 Hz,	Permeability increase to neutral red dye [34]
	Switch grass	8 kV/cm, 100 µs pulse duration, up to 5000 pulses	Permeability increase to neutral red dye [34]
Biofuel production	Yeast: in *Saccharomyces cerevisiae,* a major industrial fermentation organism	2.7 kV/cm, 15 × 10^3^ µs pulse duration, single pulse	Transformation efficiencies of 10^7^ transformants/µg of plasmid DNA were achieved, providing exciting opportunities for high-throughput genetic engineering of strains for biofuel fermentation [84]
	Microalgae: *A. protothecoides*	34 kV/cm, 1 µs pulse duration, ~0.75 MJ/kg	Cell rupture and release 15 % of algae dry weight to the medium [157]
	*C. vulgaris*	3 kV/cm, 2 × 10^3^ µs pulse duration, 30 pulses, flow rate = 1 ml/s	Protein extraction yields: 3.5 µg protein/100 µl solution (10^7^ cells/ml) [131]
	*N. salina*	6 kV/cm, 2 × 10^3^ µs pulse duration, 30 pulses, flow rate = 150 µl/s	Protein extraction yields: 5 µg protein/100 µl solution (10^8^ cells/ml) [131]
	Biogas: waste activated sludge and pig manure	10 kWh/m^3^	Increased biomethane production by 80 % for pig manure and 100 % for WAS after 25–30 days [146]

Expected future developments on the electrobiorefineries will aim at valorisation of the whole biomass feedstock. There exist some examples of PEF application for the valorisation of the whole grape (including pulp, mash, stem, skins, and seeds) [150], sugar beet (including cossettes, pulp, and tails) [150], rapeseed (including hulls, press cake, stem and leafs) [111, 151]. More examples can be expected in future for the valorisation of different crops, wood biomass, yeasts, and marine by-products. However, it is important to remember that the interpretation of results can vary widely depending on the PEF and other parameters used in the experiments. Today, authors often do not report all PEF parameters, thus making it difficult to compare different processes and results. More detailed and consistent reporting of all applied process parameters is required for continuous advancement of the field.

Selective extraction of intracellular compounds seems to be one of the most interesting features of the moderate PEF treatment for the production of high-value products from the biorefineries. Different examples of extraction selectivity from electroporated cell tissue are presented in the literature, including sucrose extraction from sugar beet, colorants extraction from grapes and red beet, polyphenols extraction from green biomass, and lipids extraction from microalgae, among others [152]. We can expect that extraction selectivity will be more explored for the future biorefineries to simplify and minimize the downstream purification operations.

For the conversion of lingo-cellulosic biomass to fermentable sugars, an efficient pretreatment strategy includes (1) disrupting and removing the cross-linked matrix of lignin and hemicelluloses that embeds the cellulose fibers, (2) disrupting hydrogen bonds in crystalline cellulose, and (3) increasing the porosity and surface area of cellulose for subsequent enzymatic hydrolysis. Conventional thermo-chemical operations (such concentrated acid hydrolysis, acidic steam explosion) are severe, high energy-consuming, costs and have negative environmental impact. Severe pretreatment conditions are also resulting in sugar degradation and inhibitor formation. It is expected that PEF treatment may contribute to better fractionation of lingo-cellulosic biomass (for e.g., its delignification [153]) and to the decrease of severity of conventional biomass conversion. The PEF treatments may also intensify cellulose enzymatic hydrolysis, which is actually slow because of low or modest cellulose digestibility. It is important to emphasize, however, that different from the PEF effects on the cell plasma membranes, where the theory of mechanisms—electroporation exist, the effects of PEF on extracellular matrix fibers are mostly observational [32, 35, 154]. Future theoretical work is needed to explain the effects of pulsed electric fields on lignocellulosic structures.

Other biorefinery applications of PEF exist. For instance, it was shown recently that PEF treatment can enhance cellular division of *S.cerevisiae* [155]. PEF may accelerate the fermentation activity, it can also influence on the methanization and can accelerate anaerobic digestion and biogas production. As the biorefineries translation to industry critically depends on the energy efficiency, PEF technologies provide a unique opportunity to reduce the energy expenditures of biorefineries with selective targeting of the cell membranes.

In recent years, advances in synthetic biology and metabolic engineering promise to revolutionize biofuels and biorefinery industry [156]. Yet, the delivery of large DNA products into the cells for the assembly of large metabolic networks is still challenging [156]. Electroporation technologies could serve as chemical-free tools for the genomic editing of entire metabolic networks by delivering multiple products into the cell with minimal levels of cell death.

Majority of the examples described in relevant scientific literature are carried out on laboratory scale batch units. Hence, in view of future industrial exploitation of this treatment, experimental work on the products of interest should be planned at pilot plant and industrial scale, to evaluate from an economical and environmental point of view the advantages of PEF-assisted processes in comparison to current technologies. Robustness tests are needed for all elements of the devices to be used, as very few long-term data are available on the performance of the systems under continuous operation in industrial facilities [37]. The other avenue of future work is to optimize PEF treatment and process parameters for each specific application. At the same time, it will be of crucial importance to gain insight on the mechanisms of electroporation and cell responses to PEF treatment, in order to be able to design and optimize the processes before scaling up.

## Abbreviations

PEF: pulsed electric fields
TMV: transmembrane voltage
pDNA: plasmid deoxyribonucleic acid
MD: molecular dynamics
LC: electric circuit consisting of an inductor (L) and a capacitor (C) connected together
PBR: photo-bioreactor
*Z*_p_: cell disintegration index
DW: dry weight
PI: propidium iodide
LY: lucifer yellow
EDTA: ethylene diamine tetra acetic acid
DTT: dithiothreitol
GUS: β-glucuronidase
CAT: chloramphenicol acetyltransferase
GAPDH: glyceraldehyde-3-phosphate dehydrogenase
PGK: 3-phosphoglycerate kinase
HK: hexokinase
BSA: bovine serum albumin
GAE: gallic acid equivalent
SDS-PAGE: sodium dodecyl sulfate polyacrylamide gel electrophoresis
